# Targeted Therapy in Biliary Tract Cancers

**DOI:** 10.1007/s11864-015-0366-0

**Published:** 2015-08-13

**Authors:** Amartej Merla, Kenneth G. Liu, Lakshmi Rajdev

**Affiliations:** Department of Medical Oncology, Montefiore Medical Center, 1695 Eastchester Road, 2nd Floor, Bronx, NY 10461 USA

**Keywords:** Biliary tract cancer, Targeted therapy, EGFR, VEGF, Trial

## Abstract

A paradigm shift towards molecular-based, personalized cancer therapeutics has occurred in recent years and a number of targeted drugs have emerged. Various targeted therapies like erlotinib, trastuzumab, and cetuximab have been approved in lung, breast, and colon cancers, respectively. Numerous clinical trials involving targeted drugs in biliary tract cancers are currently in progress, though none have been approved for this disease. Biliary tract cancers are divided into separate entities both anatomically and in terms of pathogenesis but are grouped together in most trials given their rarity. Combination chemotherapy involving cisplatin and gemcitabine is the current standard of care in the metastatic setting. In this review, we will discuss the various molecular pathways implicated in biliary tract cancers and potential therapeutic targets.

## Classification

Biliary tract cancers (BTC) are a heterogeneous group of cancers that are clinically and genetically divergent. BTC can be divided anatomically into gallbladder cancers (GBC), intrahepatic cholangiocarcinomas (IHCC), hilar cholangiocarcinomas (HCC), and extrahepatic cholangiocarcinomas (EHCC) [1]. HCC, also called Klatskin’s tumors as they were first described by Gerald Klatskin in 1965, are tumors that arise at the bifurcation of the common hepatic duct [2]. The American Joint Commission on Cancer (AJCC) 7th edition classified them as different types with separate staging systems, though the systemic management options at present remain similar. Histologically, BTC are classified as adenocarcinoma (90 % of cases), papillary, mucinous, clear cell, signet-ring cell, adenosquamous, squamous, small cell, and undifferentiated carcinomas [3]. The exact molecular mechanisms leading to BTC remain unknown.

## Epidemiology

BTC are rare malignancies with poor prognosis, with GBC being the most common among all BTC [4]. Population-based data from the National Cancer Institute’s Surveillance, Epidemiology, and End Results (SEER) program estimated 7865 newly diagnosed cases of GBC from 1992 to 2009 in the USA. Incidence is higher in females, and higher incidence rates are seen in blacks and Asians compared to whites [5]. Mortality rates are highest in Chile, India, and Eastern Europe, suggesting geographical and racial differences [6, 7]. For IHCC and EHCC, incidence rates in the USA are 0.88 per 100,000 and 0.72 per 100,000, respectively, based on data from the National Center for Health Statistics [8]. The incidence of IHCC worldwide is increasing, while that of EHCC appears to be decreasing [9, 10].

Risk factors for BTC include parasitic infections by liver flukes, gallstones, diabetes mellitus, obesity, alcohol, inflammatory bowel disease, bile duct cysts, smoking, and hepatitis B and C [11–15]. The presence of gallstones is by far the most important risk factor. The exact mechanism by which gallstones cause carcinogenesis is unknown but is probably due to persistent inflammation leading to dysplasia and accumulation of loss of heterozygosity at various tumor suppressor genes [16, 17]. The presence of gallbladder adenoma has also been proposed as a risk factor [18].

## Prognosis

GBC have the worst prognosis among all BTC. For resectable GBC, the median overall survival (OS) differs somewhat depending on the actual study. A population-based analysis using SEER data concluded that median OS for resectable GBC is only 16 months [19]. For IHCC, OS after resection is 33 months, and the 1-, 3-, and 5-year OS estimates are 82.3, 47.1, and 32.9 %, respectively. Another study reported OS of 18 months after R0 resection for node-positive disease [20, 21]. Five to ten percent of patients were alive 5 years after being diagnosed with advanced IHCC [10]. For surgically resectable HCC, the median OS is 30 months in patients undergoing combined partial hepatic resection and 24 months in those having bile duct resection only [22]. In contrast, despite an increase in treatment options, locally advanced or metastatic BTC have median OS approaching only 1 year, illustrating the need for better therapy [23].

## Chemotherapy

Treatment of early BTC is surgery, which offers potential cure. In locally advanced HCC, neoadjuvant chemoradiation followed by liver transplantation across 12 centers in the USA was shown to improve 5-year recurrence-free survival to 65 % [24]. Adjuvant therapy remains controversial and no regimen has yet been accepted as a standard. Only about 10 % of BTC are operable at the time of diagnosis. For advanced, inoperable BTC, systemic therapy is the only option. Poor prognostic factors after resection include the presence of lymph node metastases, positive margins, and poor differentiation [25, 26].

The use of chemotherapy over best supportive care was first supported in a clinical trial comparing combination of 5-fluorouracil and etoposide vs. best supportive care. The median OS in the chemotherapy group was 6 months, compared to only 2.5 months in the supportive care group [27].

The current standard of care in the metastatic setting is based on the ABC-01 and ABC-02 trials from the UK [23, 28]. ABC-01, a phase II clinical trial, demonstrated improvement in 6-month progression-free survival (PFS) favoring cisplatin and gemcitabine compared to gemcitabine alone (8.0 vs. 4.0 months) [28]. ABC-02, a phase III trial, showed an improvement in OS, with combination therapy of cisplatin and gemcitabine (11.7 months) compared to gemcitabine alone (8.1 months) (hazard ratio (HR) 0.64, 95 % confidence interval (CI) 0.52–0.80; log rank *p* < 0.001) [23]. The Japanese BT-22 trial produced results similar to the ABC-02 trial, in which 84 patients were randomized to either gemcitabine alone or to the combination of cisplatin and gemcitabine [29]. A subsequent meta-analysis concluded that BTC patients treated with gemcitabine combined with platinum agents (cisplatin or oxaliplatin) have better outcomes compared to those not treated with this regimen [30].

## Targeted therapy

Recent studies revealed multiple clinically targetable mutations in BTC. Many molecular pathways are implicated in carcinogenesis, and agents targeting these pathways have shown some efficacy in BTC cell lines [31, 32]. In a recent presentation on comprehensive genomic profiling of biliary tract cancers by Ross et al. at the 2015 Gastrointestinal Symposium, multiple genomic alterations and tumor-specific differences were noted. Next-generation sequencing was performed on 554 BTC specimens. IHCC, EHCC, and GBC share genomic alterations in cell cycle regulation (CDKN2B) and chromatin remodeling (ARID1A). IHCC harbor FGFR fusions, IDH1/2 substitutions, BRAF substitutions, and MET amplification with a low KRAS mutation frequency. EHCC and GBC both have ERBB2 amplifications and PIK3CA/mTOR pathway alterations, but KRAS mutation frequencies are high in EHCC and low in GBC [33•]. Whole exome and targeted gene sequencing in earlier studies of GBC also revealed frequent mutations in TP53, KRAS, and ERBB3 [34–36]. Microsatellite instability (MSI), which is well known in the pathogenesis of colon cancer, does not appear to play a role in BTC [37]. Various targeted agents for BTC have been tested in several phase I and II clinical trials (Table 1).

**Table 1:** Clinical trials incorporating targeted agents

Author (year)	Agent	Phase	Line	Number	ORR (%)	PFS	OS
Agents targeting the EGFR pathway							
Philip (2006) [42]	Erlotinib	II	1st/2nd	42	8	2.6 months	7.5 months
Lee (2012) [44••]	GemOx/erlotinib vs. GemOx	III	1st	135 vs. 133	30 vs. 16	5.8 vs. 4.2 months	9.5 vs. 9.5 months
Gruenberger (2010) [45]	GemOx/cetuximab	II	1st	30	63	8.8 months	15.2 months
Malka (2014) [46•]	GemOx/cetuximab vs. GemOx	II	1st	76 vs. 74	24 vs. 23	6.1 vs. 5.5 months	11.0 vs. 12.4 months
Chen (2015) [47•]	GemOx/cetuximab vs. GemOx	II	1st	62 vs. 60	27 vs. 15	6.7 vs. 4.1 months	10.6 vs. 9.8 months
Rubovszky (2013) [48]	Gem/Cape/cetuximab	II	1st	34	18	34.3 weeks	62.8 weeks
Hezel (2014) [49]	GemOx/panitumumab	II	1st	31	45	10.6 months	20.3 months
Leone (2015) [50]	GemOx/panitumumab vs. GemOx	II	1st	45 vs. 44	24.4 vs. 18.2	7.7 vs. 5.5 months	9.5 vs. 9.9 months
Jensen (2012) [51]	GemOx/Cape/panitumumab	II	1st	46	33	8.3 months	10.0 months
Sohal (2013) [52]	Gem/Irino/panitumumab	II	1st	35	69	9.7 months	12.9 months
Agents targeting the EGFR/HER2 pathway							
Ramanathan (2009) [56]	Lapatinib	II	1st/2nd	17	0	1.8 months	5.2 months
Agents targeting the VEGF pathway							
Zhu (2010) [59]	GemOx/bevacizumab	II	1st/2nd	35	40	7.0 months	12.7 months
Iyer (2015) [60]	Gem/Cape/bevacizumab	II	1st	50	24	8.1 months	11.3 months
Valle (2014) [63•]	GemCis/cediranib vs. GemCis/placebo	II	1st	62 vs. 62	43 vs. 19	7.7 vs. 7.4 months	14.1 vs. 11.9 months
Bengala (2010) [66]	Sorafenib	II	Any	46	2	2.3 months	4.4 months
El-Khoueiry (2012) [67]	Sorafenib	II	1st	31	0 (6 % unconfirmed PR)	3 months	9 months
Moehler (2014) [68]	Gem/sorafenib vs. Gem/placebo	II	1st	52 vs. 50	14 vs. 10	3.0 vs. 4.9 months	8.4 vs. 11.2 months
Lee (2013) [70]	GemCis/sorafenib	II	1st	39	12	6.5 months	14.4 months
Krege (2014) [69]	GemCis/sorafenib vs. GemCis/placebo	II	1st	40 vs. 49	52.5 vs. 47	6.3 vs. 6.1 months	11.3 vs. 10.6 months
Yi (2012) [72]	Sunitinib	II	2nd	56	8.9	1.7 months	4.8 months
Agents targeting the EGFR/VEGF pathway							
Lubner (2010) [43]	Bevacizumab/erlotinib	II	1st	53	12	4.4 months	9.9 months
Santoro (2015) [73]	Vandetanib vs. Gem/vandetanib vs. Gem/placebo	II	1st	59 vs. 58 vs. 56	3.6 vs. 19.3 vs. 13.5	105 vs. 114 vs. 148 days	228 vs. 284 vs. 307 days
Agents targeting the MEK pathway							
Bekaii-Saab (2011) [78]	Selumetinib	II	1st/2nd	28	12	3.7 months	9.8 months
Finn (2012) [79]	Binimetinib	I	1st/2nd	28	8	NR	NR
Lowery (2015) [80]	GemCis/binimetinib	I	1st	12	50	6.4 months	9.1 months
Agents targeting the mTOR pathway							
Buzzoni (2014) [84]	Everolimus	II	2nd	39	5.1	3.2 months	7.7 months
Yeung (2014) [85]	Everolimus	II	1st	27	12	6.0 months	9.5 months

*GemOx* gemcitabine/oxaliplatin, *Cape* capecitabine, *Irino* irinotecan, *GemCis* gemcitabine/cisplatin, *NR* not reported

## ErbB family signaling pathway

There are four distinct receptors that belong to the ErbB family of receptor tyrosine kinases: epidermal growth factor receptor (EGFR, also known as ErbB-1/HER1), ErbB-2 (neu, HER2), ErbB-3 (HER3), and ErbB-4 (HER4). The intracellular tyrosine kinase domain of ErbB receptors is highly conserved, while the extracellular domains are less conserved among the four receptors, suggesting different specificities in ligand binding. ErbB receptors are important in the development of different organs, and the interaction of ErbB receptors and their ligands are associated with tumorigenesis [38]. EGFR and HER2 inhibitors tested in BTC cell lines in combination with gemcitabine have shown promising activity [31], and they are being investigated in clinical trials, either alone or in combination with chemotherapy (Table 2).

**Table 2:** Ongoing clinical trials incorporating targeted agents

Drug	Class	Phase	Arms	NCT number
Cetuximab	EGFR antibody	II	Gemcitabine + oxaliplatin with or without cetuximab	NCT01267344
Afatinib	EGFR inhibitor	I	Cisplatin + gemcitabine + afatinib (single arm)	NCT01679405
Cediranib	VEGF inhibitor	II	FOLFOX with cediranib (single arm)	NCT01229111
Regorafenib	Multikinase inhibitor	II	Regorafenib (single arm)	NCT02053376
Regorafenib	Multikinase inhibitor	I/II	Gemcitabine + oxaliplatin + regorafenib (single arm)	NCT02386397
Regorafenib	Multikinase inhibitor	II	Regorafenib (single arm)	NCT02053376
Regorafenib	Multikinase inhibitor	II	Regorafenib (single arm)	NCT02115542
Ponatinib	Multikinase inhibitor	II	Ponatinib (single arm)	NCT02265341
Pazopanib	Multikinase inhibitor	II	Pazopanib (single arm)	NCT01855724
Pazopanib	Multikinase inhibitor	II	Pazopanib + gemcitabine (single arm)	NCT01855724
Trametinib	MEK inhibitor	II	Trametinib vs. 5-FU	NCT02042443
Trametinib	MEK inhibitor	II	Trametinib (single arm)	NCT01943864
Selumitinib	MEK inhibitor	II	Cisplatin + gemcitabine with or without selumitinib	NCT02151084
Binimetinib	MEK inhibitor	I/II	Cisplatin + gemcitabine + binimetinib (single arm)	NCT01828034
Vismodegib	Hh pathway inhibitor	I	Vismodegib (single arm)	NCT00968981
RO4929097	Notch inhibitor	I	RO4929097 (single arm)	NCT01096355
BGJ 398	FGFR inhibitor	II	BGJ 398 (single arm)	NCT02150967
Ponatinib	FGFR inhibitor	II	Ponatinib (single arm)	NCT02265341
AG-881	IDH inhibitor	I	AG 881 (single arm)	NCT02481154
AG-120	IDH inhibitor	I	AG-120 (single arm)	NCT02073994

### EGFR pathway

EGFR is commonly expressed in BTC and is found in 100 % of IHCC, 52.6 % of EHCC, and 38.5 % of GBC in one study [31]. A Japanese cohort study demonstrated that EGFR expression in IHCC is significantly associated with poor prognosis, while its presence in EHCC may represent tumor progression and invasion [39]. Because EGFR antibodies and inhibitors have been proven clinically to be efficacious for many cancers, they have also been investigated in BTC.

#### Erlotinib

Erlotinib is an orally active, potent, selective, and reversible inhibitor of EGFR. Its use in various cancers, such as lung, head, and neck cancers, are well documented. EGFR overexpression is seen in several BTC studies and has been studied as a therapeutic target [40, 41]. Several phase II trials have investigated the role of erlotinib in the management of advanced BTC. Philip et al. evaluated 42 patients with advanced BTC and showed median PFS of 2.6 months and median OS of 7.5 months. However, more than 50 % of these patients received prior therapy; thus, these patients might have worse characteristics compared to average. In addition, 7 (17 %) patients were progression free at 24 weeks and all were EGFR/HER1 positive, but the correlation between response and EGFR/HER1 status could not be measured accurately given the small sample size [42]. Lubner et al. investigated erlotinib in combination with bevacizumab, a vascular endothelial growth factor (VEGF) inhibitor. Nine patients (18 %) achieved an at best response of partial response (PR), 6 (12 %) of which had responses confirmed 4 weeks after their initial responses were observed. Median OS was 9.9 months, similar to that of historical controls [43].

In an open-label, randomized phase III study, Lee et al. assessed the efficacy of the addition of erlotinib to gemcitabine and oxaliplatin as first-line therapy for metastatic BTC. Two hundred sixty-eight South Korean patients were assigned in a 1:1 ratio to receive gemcitabine and oxaliplatin with or without erlotinib. Median PFS was 5.8 months in the chemotherapy plus erlotinib group and 4.2 months in the chemotherapy alone group (HR 0.80, 95 % CI 0.61–1.03, *p* = 0.087). OS was the same (9.5 months) for both groups (HR 0.93, 95 % CI 0.69–1.25, *p* = 0.611). Subgroup analysis of PFS in cholangiocarcinoma (HR 0.73) and GBC/ampulla of Vater (HR 0.99) cancers showed that much of the improvement in PFS came in the cholangiocarcinoma subgroup. This trial is the first phase III trial to assess a targeted therapy plus chemotherapy combination for patients with advanced BTC. However, the addition of erlotinib did not produce a significant improvement in median PFS. The study was flawed as it was not adequately powered and had a small sample size. The study was also limited to patients from South Korea and might not be generalizable to the non-Asian population [44••].

#### Cetuximab

Cetuximab, an anti-EGFR antibody, is evaluated in combination with gemcitabine/oxaliplatin chemotherapy in BTC in several phase II trials. An earlier study showed encouraging results in locally advanced or metastatic BTC patients who received gemcitabine/oxaliplatin plus cetuximab. Of the 30 enrolled patients, 10 % patients achieved CR, while 53 % achieved PR. Nine patients underwent potentially curative secondary resection after major response to therapy [45]. However, subsequent studies failed to demonstrate a similar benefit. Malka et al. recruited 150 patients with locally advanced or metastatic BTC, who were randomized to receive gemcitabine/oxaliplatin with or without cetuximab in the first-line setting. Median PFS was 6.1 months (95 % CI 5.1–7.6) with cetuximab and 5.5 months (95 % CI 3.7–6.6) without cetuximab, while median OS was 11.0 months (95 % CI 9.1–13.7) with cetuximab and 12.4 months (95 % CI 8.6–16.0) without cetuximab. Despite being well tolerated, the addition of cetuximab to gemcitabine and oxaliplatin did not improve outcomes when used as first-line treatment for patients with advanced BTC [46•]. Chen et al. investigated whether KRAS mutation status influences response to cetuximab. One hundred twenty-two patients were randomized to receive gemcitabine/oxaliplatin with or without cetuximab. Median PFS was 6.7 months with cetuximab and 4.1 months without cetuximab (*p* = 0.05), while median OS was 10.6 months with cetuximab and 9.8 months without cetuximab (*p* = 0.91). KRAS mutations, detected in 36 % of tumors, did not affect the objective response rate (ORR) or PFS [47•]. Cetuximab was also evaluated in combination with gemcitabine and capecitabine in the first-line setting, but ORR was only 17.6 %, with a median PFS of 32.3 weeks and OS of 62.8 weeks [48].

#### Panitumumab

Panitumumab is a fully humanized anti-EGFR antibody. Given its role in KRAS wild-type colorectal cancers, it has also been studied in the first-line setting in advanced BTC phase II trials. Hezel et al. evaluated gemcitabine and oxaliplatin in combination with panitumumab in KRAS wild-type metastatic BTC. ORR was 45 %, median PFS was 10.6 months, and median OS was 20.3 months [49]. An Italian group recently presented preliminary data in a phase II randomized, open-label trial, in which 89 patients with KRAS wild-type metastatic or unresectable BTC were randomized to receive gemcitabine and oxaliplatin with or without panitumumab. No statistical significance for median PFS and OS was noted between the two arms, though there was a trend toward improved ORR and PFS in the arm with panitumumab [50]. Jensen et al. looked at the regimen gemcitabine, oxaliplatin, and capecitabine in combination with panitumumab in KRAS wild-type advanced BTC. ORR was 33 % and the disease control rate was 86 %. Median PFS was 8.3 months and median OS was 10.0 months. The treatment was overall tolerable with some EGFR-related skin adverse events [51]. Sohal et al. reported data involving yet a different regimen, gemcitabine and irinotecan in combination with panitumumab. A promising ORR of 69 % was noted, though median PFS of 9.7 months and OS of 12.9 months were relatively similar to previous trials [52]. However, given that these phase II trials had small sample sizes, the role of panitumumab in biliary tract cancers remains to be defined.

### HER2 pathway

HER2 belongs to the ErbB family of receptor tyrosine kinases. It is overexpressed in about 10 % of GBC and 26.3 % EHCC in one study [31]. HER2 was first studied in breast carcinoma, and its overexpression in human breast carcinomas is associated with a more aggressive course of disease [53]. In an experimental tumor model, overexpression of HER2 in the basal layer of the biliary tract epithelium resulted in GBC in 100 % of transgenic mice by 3 months of age and in other BTC at a 30 % incidence rate [54]. Agents targeting HER2 are being tested in BTC patients given its efficacy in other cancers, though none clinically proven to improve outcomes thus far.

#### Trastuzumab

Trastuzumab is a monoclonal antibody that targets the HER2 receptor and is mainly used in the treatment of HER2-positive breast cancers and HER2-positive cancers involving the gastroesophageal junction. However, its role in BTC is not well defined. In a retrospective analysis, Javle et al. evaluated BTC patients with HER2 genetic aberrations or protein overexpression who received HER2-directed therapy in combination with concurrent therapy of physician’s choice. Of the eight GBC patients who received trastuzumab, CR was seen in one patient, while there were five patients with PR, one with mixed response, and one with stable disease. All five cholangiocarcinoma patients had disease progression while on trastuzumab as part of its treatment [55].

#### Lapatinib

Lapatinib is an orally administered, dual tyrosine kinase inhibitor against EGFR and HER2. It was tested in a phase II study in patients with advanced BTC but found to have no response. Median PFS was 1.8 months and median OS was 5.2 months [56].

## VEGF pathway

VEGF facilitates tumorigenesis in a variety of tumors, including BTC. Its function is not just limited to angiogenesis and vascular permeability, but also mediates signaling in tumor cells [57]. It is highly expressed in BTC, but expression varies according to the tumor type. In one study, 75 % of GBC express VEGF. VEGF appears to be correlated with more advanced and metastatic stages, and its expression is associated with poor prognosis [58].

### Bevacizumab

Bevacizumab is a recombinant humanized monoclonal antibody directed against VEGF. It has activity in various solid tumors in the metastatic setting, including colorectal, lung, breast, renal, and ovarian. In addition to the bevacizumab plus erlotinib trial described by Lubner et al. [43], bevacizumab’s role in BTC has been investigated in phase II studies in combination with other agents. Zhu et al. enrolled 35 patients who were given bevacizumab followed by gemcitabine and oxaliplatin. Median PFS was 7.0 months. FDG-PET scans demonstrated a significant decrease in maximum standardized uptake value (SUV (max)) after only 2 cycles of treatment [59]. Iyer et al. investigated yet another regimen and recently presented data looking at gemcitabine, capecitabine, and bevacizumab in patients with advanced BTC in the first-line setting. Median PFS of 8.1 months and OS of 11.3 months were similar to standard therapy [60].

### Cediranib

Cediranib is a potent inhibitor of the VEGF receptor tyrosine kinases and also with some activity against platelet-derived growth factor (PDGF) receptors and c-Kit [61]. Given that VEGF is overexpressed in BTC and VEGFR1 and VEGFR2 are overexpressed in adjacent endothelial cells, cediranib has also been investigated in a recent phase II study [58, 62]. Subjects were randomized to receive cisplatin and gemcitabine plus either cediranib or placebo. No statistically significant difference in median PFS was observed: 7.7 months (95 % CI 6.3–9.3) in the cediranib arm vs. 7.4 months (95 % CI 5.7–8.6) in the placebo arm. There was a trend towards longer OS in the cediranib arm (14.1 months, 95 % CI 10.2–16.0) compared to the placebo arm (11.9 months, 95 % CI 9.2–13.4). However, it did appear to improve the ORR in this trial: 43 % in the cediranib arm compared to 19 % in the placebo arm [63•]. A phase II trial investigating cediranib in combination with modified FOLFOX6 in advanced BTC is now underway [64].

### Sorafenib

Sorafenib is an oral multikinase inhibitor that inhibits cell surface tyrosine kinase receptors, including VEGF receptors and PDGF receptor-β, and blocks downstream intracellular serine/threonine kinases, such as Raf-1, wild-type, and mutant B-Raf, involved in tumor cell proliferation and angiogenesis [65]. It has been shown to be effective in hepatocellular carcinoma and renal cell carcinoma, but the same has not been demonstrated in advanced BTC. In one study, ORR was only 2 %, median PFS was 2.3 months, and median OS was 4.4 months [66]. Another trial involving sorafenib was terminated early given that it failed to meet the primary objective or response probability. No objective responses were observed. Median PFS was 3 months and median OS was 9 months [67].

Other trials have evaluated sorafenib in combination with standard chemotherapy. Moehler et al. investigated gemcitabine with either sorafenib or placebo in the first-line setting. However, longer median PFS and OS were actually seen in the gemcitabine plus placebo arm [68]. A randomized, double-blinded, multicenter phase II trial by Krege et al. comparing a combination of gemcitabine, cisplatin with either sorafenib or placebo found no significant difference in PFS and OS between the two arms [69]. Finally, Lee et al. compared cisplatin, gemcitabine, and sorafenib to historical data and showed no improvement in efficacy, but there was an increase in toxicity [70].

### Sunitinib

Sunitinib is another oral small molecule inhibitor that targets multiple intracellular and receptor protein kinases, including VEGF and PDGF receptors, c-Kit, and rearranged during transfection (RET). It is approved for gastrointestinal stromal tumor and renal cell carcinoma [71]. In a recent phase II study, Yi et al. investigated the role of sunitinib as a second-line treatment in advanced BTC. Only marginal efficacy was demonstrated. ORR was 8.9 % and median PFS was 1.7 months [72].

### Vandetanib

Vandetanib is an oral multikinase inhibitor that targets VEGF, EGFR, as well as the RET kinase. Its role in the treatment of thyroid cancer has been well documented. In an Italian (VanGogh) study, 165 BTC patients who had not received any prior chemotherapy were randomized into three groups: vandetanib monotherapy (V), vandetanib plus gemcitabine (V/G), and gemcitabine plus placebo (G/P). Median PFS was 105 days (95 % CI 72–155), 114 days (95 % CI 91–193), and 148 days (95 % CI 71–225), while median OS was 228 days (95 % CI 190–364), 284 days (95 % CI 213–359), and 307 days (95 % CI 254–523) for V, V/G, and G/P, respectively. This study did not demonstrate any superiority of vandetanib alone or in association with gemcitabine when compared with gemcitabine alone [73•].

## MEK pathway

In addition to targeting growth factor receptors, recent research has also focused on interfering with various signaling pathways essential to the regulation of cellular processes. These include the RAS/RAF/mitogen-activated protein kinase kinase (MEK)/extracellular signal-related kinase (ERK) signaling pathway, which is activated by a diverse group of extracellular signals, such as growth factor receptors and cytokines. Activated RAS starts a phosphorylation cascade involving RAF kinase, MEK1/MEK2, and ERK1/ERK2 [74]. Phosphorylated ERKs form homodimers and translocate to the nucleus to perform important cellular functions [75]. In preclinical studies, CI-1040, a MEK inhibitor, has been investigated in a panel of human cancer cell lines and showed broad activity, particularly in cell lines harboring the BRAF mutation [76]. One study reported that BRAF mutations were identified in 22 % of cholangiocarcinoma, suggesting the potential role of MEK inhibitors in the management of BTC [77].

Selumetinib is a second-generation, potent, uncompetitive inhibitor of MEK1/2. It was investigated in a phase II study for patients with advanced BTC. Median PFS was 3.7 months and OS was 9.8 months. The absence of phosphorylated ERK staining was associated with a lack of response [78]. Binimetinib, another potent, uncompetitive inhibitor of MEK1/2, was investigated in phase I trials both alone and in combination with standard chemotherapy, with encouraging activity and tolerable safety profile [79, 80]. Phase II studies exploring this drug in BTC are ongoing (NCT02151084, NCT01828034).

## PIK3CA/PTEN/AKT/mTOR pathway

The phosphatidylinositol 3-kinase (PIK3CA)/phosphatase and tensin homologue (PTEN)/AKT/mammalian target of rapamycin (mTOR) pathway is important for cell growth and survival. Abnormal activation and genetic mutations in this pathway predispose to the development of many cancers, and hence, this pathway has gained importance in recent years as a target for drug development. A study revealed that 12.5 % of patients with GBC have activating mutations in PIK3CA [81]. Deregulation of this pathway has been shown to induce GBC in normal gallbladder epithelial cells [82]. Buparlisib (BKM120), a PI3K inhibitor, is being tested in various phases of clinical trials in several malignancies either as a single agent or combined with other targeted agents or conventional chemotherapy.

mTOR is upregulated in many cancer types and is currently indicated for the treatment of advanced breast cancer, neuroendocrine tumors of pancreatic origin, and renal cell carcinoma [83]. In a phase II Italian study, 39 patients with locally advanced, metastatic, or recurrent BTC progressing despite previous chemotherapy received everolimus, an inhibitor of mTOR. ORR was only 5.1 %, median PFS was 3.2 months, and median OS was 7.7 months [84]. In a phase II Australian study, everolimus was administered in the first-line setting in advanced BTC. Preliminary results were recently presented, with ORR 12 %, median PFS 6.0 months, and median OS 9.5 months [85]. Larger studies are necessary to clarify the efficacy of mTOR inhibitors in the treatment of BTC.

Heat shock protein (HSP90) is a chaperone protein that helps maintain structural integrity and function of specific protein targets involved in cell cycle. It has antiapoptotic activity and can target multiple pathways with promising role in many malignancies. The combination of HSP90 inhibitor and PI3K/mTOR inhibitor was synergistic, inducing cell death in preclinical models of cholangiocarcinoma [86].

## Hedgehog pathway

The Hedgehog signaling pathway is involved in numerous developmental processes including cell patterning, cell fate, proliferation, survival, and differentiation during early development [87]. This pathway is dormant in most adult tissues, but its activation has been implicated in carcinogenesis. The components of the Hedgehog pathway include three secreted ligands (Sonic Hh, Indian Hh, and Desert Hh), a negative regulatory receptor (Patched [PTCH]), a positive regulatory receptor (smoothened [SMO]), and glioma-associated oncogene transcription factors. Nevoid basal cell carcinoma syndrome consisting of multiple basal cell carcinomas is associated with germline loss of function of mutation in PTCH. PTCH 1 and 2 mutations are also found in 30 % of sporadic basal cell carcinomas [88]. Vismodegib, a small molecule that inhibits the Hedgehog pathway, is approved in basal cell carcinoma and is being studied in phase I and II trials in solid tumors [89]. Blockage of the Hedgehog pathway has been shown to decrease survival and proliferation of cancer cells in cholangiocarcinoma cell lines [90].

## Notch pathway

The Notch pathway is another attractive target in cancer therapy. It is essential for tumor angiogenesis and is needed to establish and maintain stem cell population. It has an established role in hematologic malignancies [91]. Notch signaling can be oncogenic or tumor suppressive depending on the cellular context. Mammals have four notch receptors (1–4) and five ligands. Alteration of Notch pathways in solid and liquid tumors can lead to oncogenesis [92].

A study revealed that aberrant expression of Notch receptors 1 and 3 may play a role in the cancer progression of BTC [93]. RO-4929097 (a notch signaling inhibitor) is being tested in various phase I and II trials in solid tumors (Table 2). Further research is needed to determine if this pathway can be used as a potential target in BTC.

## Immunotherapy

Tumors suppress the immune system in the tumor microenvironment and also systemically. The immune system responds to cancer by reacting against tumor-specific or tumor-associated antigens (TAA). Major developments in immunological techniques have helped us discover that the immune system does recognize cancer antigens. Many TAA used in cancer therapeutics have been developed in recent years. Passive immunity is using monoclonal antibodies against tumor antigens or infusion of tumor-specific autologous T cells. Induction of active immunity by vaccination, which boosts tumor-specific antibodies and T cells, is being studied [94].

MUC1 is a large membrane glycoprotein that is identified as a tumor antigen. The MUC1 peptide vaccine has been evaluated in pancreatic and BTC in two separate trials. Yamamoto et al. evaluated eight eligible patients with metastatic disease. Seven had progressive disease and one had stable disease with a tendency for increased circulating anti-MUC1 IgG antibody after vaccination [95]. Lepisto et al. conducted a phase I/II trial using MUC1 peptide-loaded dentritic cells (DC) vaccine in the adjuvant setting in BTC or pancreatic cancer. The vaccine was well tolerated with no significant toxicities. Twelve patients were enrolled and four of them were alive at 4 years [96].

Subsequently, Shimizu et al. conducted a phase I trial on 36 patients with IHCC after resection. Patients were vaccinated with autologous tumor lysate pulsed DC plus ex vivo activated T-cell transfer. Median PFS and OS were 18.3 and 31.9 months in patients receiving adjuvant immunotherapy and 7.7 and 17.4 months in the group receiving surgery alone (*p* = 0.005 and 0.022, respectively) [97]. Further randomized controlled trials are needed to study the effects of immunotherapy in BTC.

## Others

The fibroblast growth factor receptor (FGFR) pathway is involved in cellular migration, proliferation, survival, and differentiation. Various mutational profiling studies in cholangiocarcinoma have detected genetic alterations in the FGFR pathway, exclusively in IHCC, and are shown to be associated with a more indolent course [98, 99]. FGFR2 fusions occur in 13.6 % of IHCC [100]. BGJ 398, an oral FGFR inhibitor, is being evaluated in a phase II trial in patients with FGFR2 gene fusion/translocation [101]. A number of other clinical trials involving FGFR small molecule inhibitors in BTC are in progress (Table 2).

Isocitrate dehydrogenase (IDH) catalyzes the oxidative decarboxylation of isocitrate to α-ketoglutarate (α-KG). α-KG, a substrate for multiple enzymes, is important for cellular response to oxidative stress. *IDH* mutation results in accumulation of D-2-hydroxyglutarate, predisposing cells to malignant transformation. These mutations are commonly found in gliomas and acute myeloid leukemias. A study reported IDH 1 and 2 mutations in IHCC (9 of 40, 23 %), but none in EHCC and GBC cases [102•]. The effect of IDH inhibitors is currently being evaluated in clinical trials involving solid tumors such as cholangiocarcinoma that harbor these mutations [103, 104].

Ribonucleotide reductase is an enzyme involved in DNA synthesis. 3-Aminopyridine-2-carboxaldehydethiosemicarbazone (3-AP), a new, potent, small molecule inhibitor of ribonucleotide reductase was tested in combination with gemcitabine in BTC. ORR was seen in 3 of the 23 tested patients (13 %) [105].

GBC are twice more common in females and expression of estrogen receptors in GBC has been shown. Targeting estrogen receptors could have some role in the treatment, though the exact mechanism of how these receptors promote GBC is unknown [106].

## Conclusion

BTC are rare malignancies in western countries but very prevalent in northern India and Chile. GBC, the most aggressive cancer among all, is categorized as an orphan disease. Despite recent advances in our knowledge on the pathogenesis of BTC at the molecular level, prognosis remains poor. The Cancer Genome Atlas (TCGA) helps identify genomic changes in specific tumor types and develop an integrated picture of commonalities and differences across tumor lineages [107]. BTC are currently being investigated in TCGA. Understanding the genomic alterations may help us better understand the pathogenesis and identify targetable mutations. Finally, the difficulty in the classification, molecular heterogeneity, and rarity of this disease makes clinical trials more challenging. Detection of tumors at earlier stages and improvement in surgical techniques are needed, but advancement of systemic treatment is also necessary. Targeted drugs against ErbB, VEGF, PI3K/AKT, MEK/ERK, Hedgehog, and Notch pathways are being investigated in BTC (Table 2). Enrollment of patients in clinical trials and close collaboration among international cancer organizations may lead to better outcomes in this aggressive disease.
